# Functional role of *SETD2, BAP1, PARP-3* and *PBRM1* candidate genes on the regulation of *hTERT* gene expression

**DOI:** 10.18632/oncotarget.18712

**Published:** 2017-06-27

**Authors:** Hannah Linne, Hemad Yasaei, Alison Marriott, Amanda Harvey, Kefah Mokbel, Robert Newbold, Terry Roberts

**Affiliations:** ^1^ College of Health and Life Sciences, Department of Life Sciences, Biosciences, Brunel University London, Middlesex, UK; ^2^ Institute of Environment, Health and Societies, Brunel University London, Middlesex, UK; ^3^ London Breast Institute, The Princess Grace Hospital, London, UK; ^4^ Current address: Dubai Genetics Centre, Dubai Health Authority, Dubai, United Arab Emirates

**Keywords:** telomerase, breast cancer, epigenetic, chromosome 3, microcell-mediated chromosome transfer

## Abstract

Narrowing the search for the critical *hTERT* repressor sequence(s) has identified three regions on chromosome 3p (3p12-p21.1, 3p21.2 and 3p21.3-p22). However, the precise location and identity of the sequence(s) responsible for *hTERT* transcriptional repression remains elusive. In order to identify critical *hTERT* repressor sequences located within human chromosome 3p12-p22, we investigated *hTERT* transcriptional activity within 21NT microcell hybrid clones containing chromosome 3 fragments. Mapping of chromosome 3 structure in a single *hTERT-*repressed 21NT-#3fragment hybrid clone, revealed a 490kb region of deletion localised to 3p21.3 and encompassing the histone H3, lysine 36 (H3K36) trimethyltransferase enzyme SETD2; a putative tumour suppressor gene in breast cancer. Three additional genes, BAP1, PARP-3 and PBRM1, were also selected for further investigation based on their location within the 3p21.1-p21.3 region, together with their documented role in the epigenetic regulation of target gene expression or *hTERT* regulation. All four genes (SETD2, BAP1, PARP-3 and PBRM1) were found to be expressed at low levels in 21NT. Gene copy number variation (CNV) analysis of SETD2, BAP1, PARP-3 and PBRM1 within a panel of nine breast cancer cell lines demonstrated single copy number loss of all candidate genes within five (56%) cell lines (including 21NT cells). Stable, forced overexpression of BAP1, but not PARP2, SETD2 or PBRM1, within 21NT cells was associated with a significant reduction in *hTERT* expression levels relative to wild-type controls. We propose that at least two sequences exist on human chromosome 3p, that function to regulate *hTERT* transcription within human breast cancer cells.

## INTRODUCTION

Cellular immortality is one of the ten hallmarks of human cancer and is an essential pre-requisite for malignant progression [1–3]. In contrast, normal human somatic cells proliferate for a limited number of population doublings before entering a permanent state of growth arrest known as replicative senescence [4, 5]. Telomeres are specialised nucleoprotein structures located at eukaryotic chromosome ends, that function to maintain the stability and integrity of chromosome structure, and play an important role in regulating cellular lifespan [6]. The reverse transcriptase ribonucleoprotein enzyme complex, telomerase, which is encoded by the *hTERT* gene, synthesizes telomeric repeat sequences at eukaryotic chromosome termini [7]. Over 90% of human tumours have been found to express telomerase, whereas the majority of normal human somatic cells do not [8]. This implies that activation of telomerase could be responsible for the unlimited replicative potential of human tumour cells. Telomerase-positive human tumour cell lines have been found to contain up to 6 molecules of cytoplasmic hTERT mRNA, whereas telomerase-negative normal cells lack detectable levels [9]. Furthermore, intron-containing pre-spliced *hTERT* mRNA can only be detected within telomerase-positive cells. This suggests that telomerase expression is regulated primarily by the rate of *hTERT* transcription. Spontaneous immortalisation of normal human somatic cells has never been found to occur *in vitro*; however, ectopic expression of *hTERT* within normal primary human fibroblasts and epithelial cells can be sufficient to confer cellular immortality [10–12]. Furthermore, inhibition of *hTERT* expression within telomerase-positive human tumour cells is associated with telomere shortening and induction of cell death or senescence pathways *in vitro* and elimination of tumour formation *in vivo* [13–15]. It is therefore thought that de-repression of *hTERT* expression is a critical step for telomerase reactivation and subsequent cellular immortalisation during human carcinogenesis.

Transcription factors, hormones, as well as epigenetic mechanisms have been found to regulate *hTERT* expression within human cells [16]. However, the precise mechanisms that confer *hTERT* de-repression during carcinogenesis remain elusive. Whole somatic cell fusion of immortal tumour cell lines with normal human cells has been found to produce telomerase-repressed hybrids with a finite replicative capacity [17]. *hTERT* expression within human tumour cells therefore behaves as a recessive trait, which suggests that loss of critical repressor sequences may be responsible for *hTERT* activation. Evidence from human monochromosome transfer studies support this notion and demonstrate that *hTERT* repressor sequences exist on chromosomes 3, 4, 6, 7, 10 and 17 [reviewed by 17, 18].

We have shown previously that introduction of a normal, intact copy of human chromosome 3 into the 21NT primary breast cancer cell line by microcell-mediated monochromosome transfer (MMCT), is associated with strong repression of telomerase and *hTERT* transcription and induction of growth arrest within the majority of hybrid clones [9, 14]. Structural mapping of chromosome 3 within telomerase-positive revertent clones revealed two regions of deletion: 3p21.3-p22 and 3p12-p21.1, thought to harbour the putative telomerase repressor sequence(s). A subsequent study showed that the chromosome 3p-encoded telomerase repressor sequence(s) mediates its function by means of transcriptional silencing of *hTERT,* in part, through chromatin remodelling of two sites within intron 2 of the *hTERT* gene [19]. Our mapping data providing evidence suggesting that *hTERT* repressor sequences may be present within a 350kb region on 3p21.2 [18–19].

In this study, we narrowed the search for chromosome 3p-encoded sequences responsible for conferring *hTERT* repression within 21NT cells by mapping the chromosome 3 structure in a single *hTERT-*repressed 21NT microcell hybrid containing a chromosome 3 fragment. We identified a 490kb region, localised to 3p21.3, which encompasses the putative breast cancer tumour suppressor gene and H3K36 trimethyltransferase known as SETD2 [20, 21]. In addition to SETD2, a review of relevant literature identified a further three candidate telomerase repressor genes that (i) are located proximal to or within the 3p21.3-p22 region, (ii) have been previously implicated in breast cancer and (iii) have been shown to play a functional role in the epigenetic regulation of target gene transcription through chromatin remodelling and/or have been implicated in *hTERT* regulation within other cancer cell types. We report that stable, forced overexpression of BAP1 within 21NT cells is associated with a significant reduction in immature *hTERT* mRNA expression levels relative to wild-type controls. Our results suggest that at least two sequences on chromosome 3p function to regulate *hTERT* transcription within human breast cancer cells.

## RESULTS

### Localization of a 490kb region on human chromosome 3p21.31 associated with repression of pre-spliced hTERT transcription in human 21NT breast cancer cells

**Figure 1:**
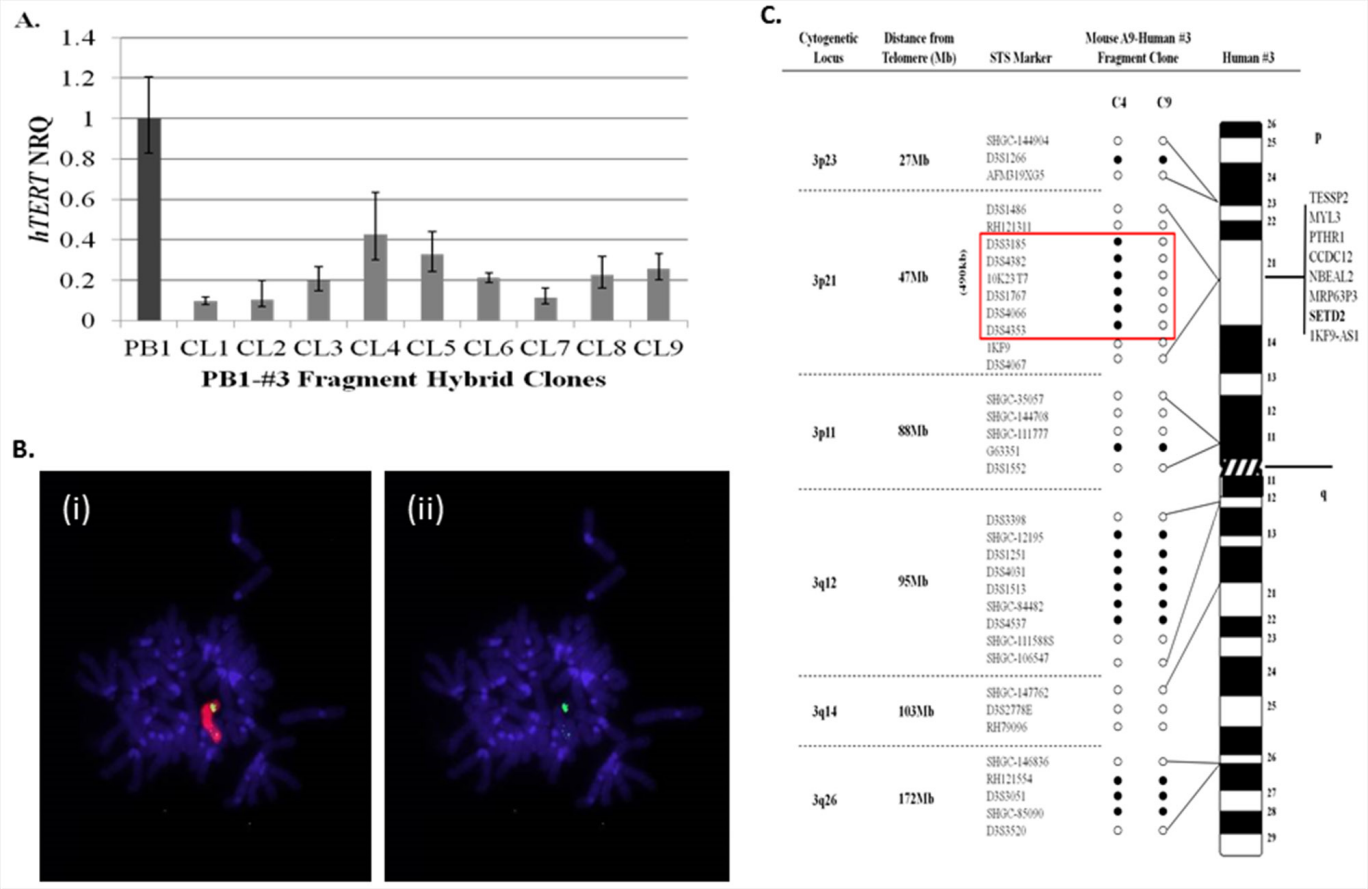
Localisation of a 490kb region on human chromosome 3p that represses *hTERT* transcription within 21NT-*hTERT* (PB1) breast cancer cells **(A)** qRT-PCR analysis of pre-spliced *hTERT* expression levels (mean ± S.E n=3) within PB1-human chromosome 3 (PB1-#3fragment) hybrid clones, relative to wild-type PB1 cells. **(B)** FISH analysis of a single mouse A9-clone 8#3fragment hybrid generated by retro-transfer of the #3 fragment from clone 8 into mouse A9 cells. Representative DAPI-stained metaphase spreads of mouse A9-clone 8#3fragment clones, hybridised with, **(i)** TexasRed-labelled total human genomic paint and **(ii)** FITC-labelled chromosome 3-specific painting probes. **(C)** Summary of chromosome 3 microsatellite sequence-tagged site (STS) analysis of two mouse A9-clone 8#3 fragment hybrids (clones 4 and 9). A list of genes located within the 490kb region of deletion observed in clone 9 is indicated. (NRQ-Normalised relative quantity) Open circle represents loss of the marker while filled circles retained the marker.

The initial aim of the study, was to narrow the search for sequences localised to human chromosome 3p that function to repress *hTERT* transcription within 21NT breast cancer cells. To achieve this, chromosome 3 fragments generated by radiation exposure of A9-Hytk3 human: mouse monochromosome hybrids, were introduced into PB1 (21NT-exo*hTERT*) cells by microcell transfer. 30 individual PB1-#3fragment hybrid clones containing chromosome 3 fragments were picked and propagated as individual clones. The hybrids were mapped using polymorphic markers spanning the whole length of human chromosome 3 (data not shown). *hTERT* quantitation was also performed on all hybrid clones using real-time qPCR (data not shown). As shown in (Figure 1A), nine of the PB1-#3fragment hybrid clones generated, exhibited a greater than 55% reduction in pre-spliced *hTERT* mRNA relative to wild-type (PB1) controls. Genotyping revealed that these clones (CL1-9) contained the smallest fragments of chromosome 3 and still retained the ability to repress *hTERT* (the other clones contained much larger fragments which would make searching for the repressor much more difficult). Some of the clones however were unstable (CL1, CL2 and CL7) and did not grow beyond a few passages. Clone CL8 proved to be the most stable when continuously passaged, therefore it was selected for further analysis by transferring it back into the mouse A9 background. The chromosome 3 fragment residing within clone 8 (CL8), which demonstrated a 77% reduction in *hTERT* mRNA levels relative to wild-type 21NT cells, was retro-transferred into mouse A9 fibroblasts by microcell transfer in order to facilitate mapping using a series of non-informative polymorphic markers. Cytogenetic analysis was carried out on the retro-transferred mouse cell line (A9-clone 8#3fragment hybrids) and revealed the presence of human chromosome material containing a single discreet region of chromosome 3 material (Figure 1B). Mapping of chromosome 3 structure within mouse A9-clone8#3 fragment hybrids using a series of polymorphic marker sequences, showed that the chromosome 3 fragment consisted of discreet regions of short arm (3p23, p21 and p11) and long arm (3q12 and q26) material (Figure 1C). Notably, one A9-clone8#3 fragment hybrid (C9) showed loss of a 490kb sequence, which maps to the 3p21.3 region. In our lab we have shown that when chromosomes are transferred into recipient cells using MMCT, a percentage of the resulting clones acquire spontaneous chromosomal deletions of specific regions [14]. Clones CL1-9 were shown to have the smallest chromosome 3 fragments which repress hTERT. When clone PB1#3CL8 was transferred back into the A9 fibroblast background, 14 clones in total were picked. Most were identical to C4, however 2 clones were obtained with spontaneous deletions within the 47Mb region at 3p21. One of these clones was C9. We considered C4 to be the clone which caused hTERT repression. Frequent loss of this 47Mb region was observed previously within telomerase-positive segregant 21NT-chromosome 3 hybrids [14, 19]. For this reason, the 3p21.3 region was considered a strong candidate to harbour the putative *hTERT* repressor sequence. Out of all six annotated genes located within this 490kb region (Figure 1C), *SETD2* has previously been shown to function as an epigenetic regulator of gene transcription through histone H3 lysine 36 (H3K36) trimethylation [22]. A putative tumour suppressive role of *SETD2* in breast cancer was reported by Sarakbi *et al.* [20], which demonstrated that SETD2 mRNA levels within malignant breast cancer tissues are significantly lower than normal breast tissue samples. Similarly, *SETD2* mRNA levels have been found to be significantly lower within tumour samples than matched adjacent non-cancerous tissue (ANCT) samples from 25 breast cancer patients [21]. These findings, together with the results presented in the present study, prompted us to investigate the functional role of SETD2 in the regulation of *hTERT* transcription within 21NT cells.

### Identification of three additional telomerase repressor candidate genes localised to human chromosome 3p21.1-21.3

Three additional candidate genes were selected for further investigation as potential telomerase repressors (see methods section for details). These were BRCA1 associated protein-1 (BAP1), polybromo 1 (PBRM1) and poly(ADP) ribose polymerase 3 (PARP-3).

**Figure 2:**
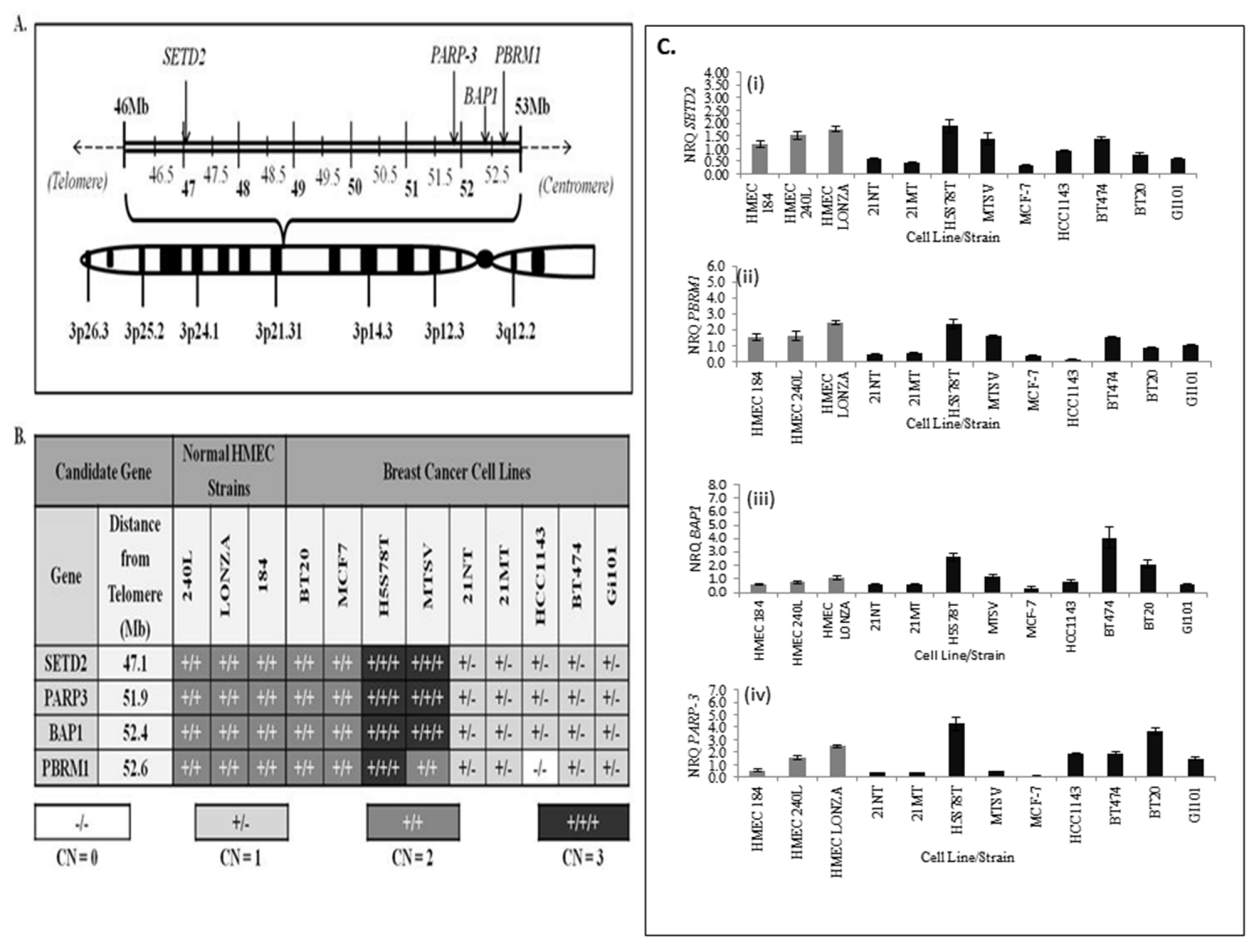
The genomic positions and gene copy numbers of *SETD2, PARP3, BAP1* and *PBRM1* within a panel of breast cancer cell lines and normal human mammary epithelial cell (HMEC) strains **(A)** Graphical representation of the approximate positions of candidate genes within the 3p21.1-p21.3 region of human chromosome 3. Genomic coordinates of all candidate genes were obtained from the National Centre of Biotechnology Information (NCBI) database. **(B)** Candidate gene copy number (CN) variation analysis of HMEC strains and a panel of nine breast cancer cell lines. **(C)** Real-time qPCR mRNA expression analysis for SETD2, PBRM1, BAP1 and PARP-3 in breast cancer cell lines and normal breast cells (HMEC’s).

In order to carry out a preliminary analysis of the putative tumour suppressive role of SETD2, BAP1, PBRM1 and PARP-3 within breast cancer cells, we carried out gene CNV analysis of all candidates within a panel of nine breast cancer cell lines relative to three normal HMEC cell strains (see Supplementary Table 1 for the origin of each cell line/strain used). Over half (56%) of breast cancer cell lines, including 21NT cells, were found to have undergone single copy loss of all candidate genes, whereas 22% of cell lines had gained a single copy, and 22% had no change in gene copy number compared with normal HMECs (Figure 2A and 2B). The SV40-immortalised breast cell line MTSV and the HCC1143 breast carcinoma cell line were found to have undergone distinct copy number alterations within the *PBRM1* locus compared with the other loci examined. These findings are consistent with Xia *et al* [23], where a homozygous deletion encompassing exons 12-17 of the *PBRM1* locus within HCC1143 cells was identified. Due to the high frequency of gene copy number loss within breast cancer cells, our results provide additional evidence that SETD2, BAP1, PBRM1 and PARP-3 may function as tumour suppressors in breast cancer cells. However, the functional consequence of this ∼5.5Mb single copy deletion on chromosome 3p21.1-3p21 within the majority of these breast cancer cell lines is unknown (Figure 2A and 2B). We next performed real-time qPCR on the breast cancer cell lines and normal breast tissues using primer sequences specific to the candidate genes SETD2, BAP1 PBRM1 and PARP-3 (Figure 2Ci-2Civ). In all cases, the level of expression of the 4 candidate genes were higher in normal tissues (HMEC’s) and lower in our chosen breast cancer cell lines 21NT and 21MT. This suggests that these genes may have tumour suppressive/telomerase repressive activity in 21NT and 21MT.

### Chromosome 3p21.1-21.3 encoded sequences BAP1, but not SETD2 PARP-3 and PBRM1, repress hTERT transcription within 21NT cells

**Figure 3:**
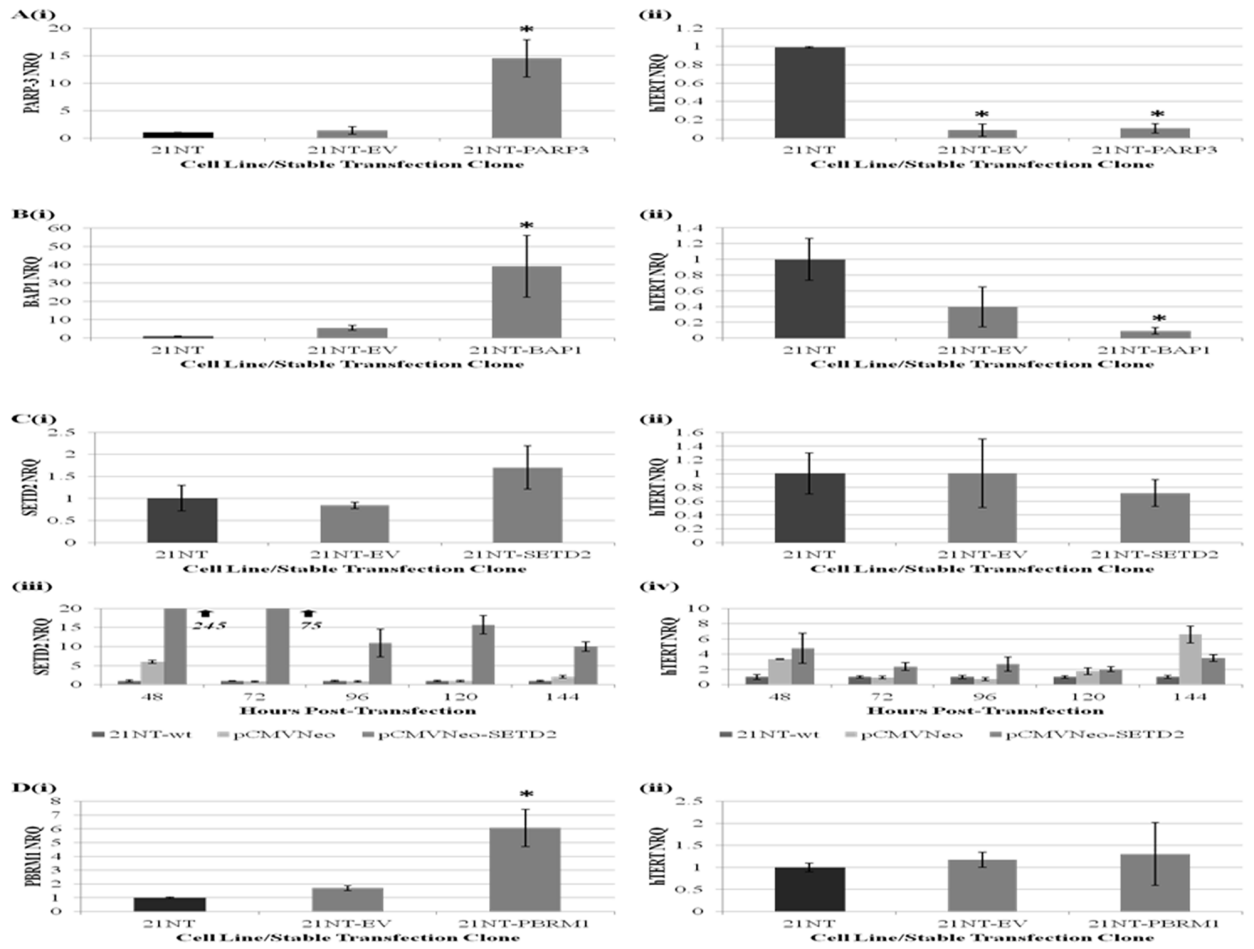
The effect of forced, stable overexpression of PARP-3, BAP1, SETD2 and PBRM1 on hTERT transcription within 21NT breast cancer cells qRT-PCR analysis of average **(Ai)** *PARP-3*, **(Bi)** *BAP1*, **(Ci)** *SETD2* and **(Di)** *PBRM1* expression levels (mean ± S.E n=3) and **(Aii, Bii, Cii, and Dii)** pre-spliced *hTERT* expression levels (mean ± S.E n=3) across five independent stable 21NT-candidate gene transfection clones and five independent stable 21NT-empty vector (EV) clones relative to parental 21NT cells (*p<0.05). Figure 3C shows **(iii)** *SETD2* and **(iv)** *hTERT* expression levels (mean ± SD) within 21NT cells 48-144 hours following transient transfection of 21NT cells with pCMVNeo (EV) and pCMVNeo-*SETD2* vector constructs, expressed relative to untreated 21NT cells. (NRQ-Normalised Relative Quantity).

In order to investigate the functional role of SETD2, BAP1, PBRM1 and PARP-3 in regulating *hTERT* transcription, we examined the effect of forced, stable overexpression of candidate genes on pre-spliced *hTERT* expression within 21NT breast cancer cells. Following positive selection and isolation of stable clones, candidate gene and *hTERT* expression levels were examined. As shown in (Figure 3A, 3B and 3D), overexpression of PARP-3 and BAP1, but not PBRM1, within 21NT cells was associated with a significant reduction in *hTERT* expression levels when compared with wild-type 21NT cells. However, for PARP-3 (and to a lesser extent BAP1) transfection with the empty vector control also decreased *hTERT* expression.

Stable 21NT-SETD2 clones were found to exhibit a 1.7-fold average increase in *SETD2* expression levels relative to untreated controls, which did not reach statistical significance. Therefore, the effect of increased SETD2 expression on *hTERT* transcriptional activity was unclear. In order to investigate this further, the effect of transient, high-level *SETD2* expression on *hTERT* transcription over the course of six consecutive days was examined. For all time points, increased *SETD2* expression was found to be associated with increased *hTERT* expression levels relative to untreated controls (Figure 3Ciii and 3Civ). Despite empty vector controls also exhibiting a substantial increase in *hTERT* expression levels at 48, 120 and 144 hours post-transfection, it is clear that increased *SETD2* expression was not associated with repression of *hTERT* transcription.

## DISCUSSION

Over 90% of human breast carcinomas and up to 75% of ductal carcinoma *in situ* (DCIS) lesions exhibit detectable levels of telomerase activity, whereas atypical ductal hyperplasia (ADH) and normal breast tissues do not [24–25]. Thus, telomerase reactivation is an early event during breast carcinogenesis that may promote tumour progression. A significant positive correlation between *hTERT* mRNA levels and telomerase activity within human breast carcinomas has also been reported, which supports the notion that *hTERT* is the critical rate-limiting component of telomerase enzyme activity [26]. Independent studies demonstrate a relationship between telomerase activity and breast tumour stage, nodal metastases and decreased disease-free survival [27–28]. To date, the key molecular mechanism(s) underlying *hTERT* transcriptional de-repression and telomerase reactivation during human breast carcinogenesis remain elusive.

Our group has demonstrated previously that introduction of a normal intact copy of human chromosome 3 into the primary breast carcinoma cell line 21NT is associated with strong repression of telomerase and *hTERT* transcription and induction of cell growth arrest within the majority of hybrids [14, 19]. We also identified three candidate regions within 3p12-p22 that were thought to harbour the critical *hTERT* repressor sequence(s) [14, 19]. In the present study, radiation-induced chromosome 3 fragments were transferred into 21NT breast cancer cells by MMCT and a single fragment that was associated with*hTERT* transcriptional repression was identified. Following MMCT-mediated retro-transfer of this fragment into mouse A9 fibroblast cells, sequence-tagged-site (STS) mapping of hybrid clones demonstrated that it was a chimeric fragment. It contained regions of chromosome 3 short and long-arm material including a 490kb sequence located within the 3p21.3-p22 region, which was implicated in *hTERT* regulation by our group previously [14, 19]. In addition, studies have shown that the 3p21-p22 region is a frequent site of loss of heterozygosity (LOH) in breast cancer, indicating the presence of important tumour suppressor genes [29–30]. In support of this, the results presented in the present study demonstrated that 56% of breast cancer cell lines had undergone single copy number loss of four candidate *hTERT-*repressor genes (*SETD2, BAP1, PBRM1* and *PARP-3*) spanning a 5.5 Mb region within 3p21.1-p21.3, which adds to the body of evidence that these genes play an important tumour suppressive role in breast cancer cells [20, 21, 26–30]. In addition, we have demonstrated that *SETD2, BAP1, PBRM1* and *PARP-3* are all down-regulated in the breast cancer cell line 21NT when compared to normal cells (HMEC), making them prime targets for tumour suppression / telomerase repression in breast cancer.

In the present study, overexpression of BAP1, but not PARP3, PBRM1 or SETD2, was associated with significant (but not complete) repression of *hTERT* transcription within 21NT breast cancer cells. This suggests that at least two sequences may function, either independently or collectively, to confer *hTERT* repression in human breast cancer cells. Primary breast tumours have been found to undergo discontinuous allele loss of multiple regions of chromosome 3p [29]. Reverse selection of 21NT-chromosome 3 hybrids that had lost the introduced chromosome 3 copy by ganciclovir (GCV) selection by our group previously [19], was associated with the restoration of an ‘open’ chromatin conformation around intron 2 of *hTERT,* but not associated with consistent re-activation of *hTERT* expression when compared to parental 21NT cells. Despite ensuring the absence of residual, exogenous chromosome 3p14.1-21.2 material within the majority of revertant clones, the presence of another repressor sequence outside of this region (perhaps within 3p21.2-p22) may have been responsible for the sustained repression of *hTERT* transcription*.* Therefore, it is possible that one chromosome 3p-encoded sequence functions to regulate the chromatin conformation around intron 2 of *hTERT,* while another sequence(s) may function to prevent binding of transcriptional activators of *hTERT* within or around this region.

In support of the notion that multiple *hTERT* repressor sequences exist on human chromosome 3p, whole somatic cell fusion of 21NT cells with two renal cell carcinoma cell lines (RCC23 and KC12), which are also known to confer *hTERT* repression when a normal chromosome 3 copy is introduced into these cell lines by MMCT, produces telomerase-negative hybrids [31–33]. A recent study has also reported that two functionally distinct *hTERT* regulatory sequences exist on human chromosome 3p within human renal cell carcinoma and oral squamous cell carcinoma cells [34].

In conclusion, the results from the present study provide evidence to suggest that BAP1 and possibly PARP-3 repress *hTERT* transcription within breast cancer cells, which supports the hypothesis that multiple sequences on human chromosome 3p may be responsible for regulating *hTERT* transcription. Despite efforts to achieve positional cloning of critical *hTERT* repressor sequences in different cancers, the key molecular players and mechanisms by which they function to repress *hTERT* transcription remain unclear. Further investigation involving high resolution transcriptome profiling and next generation sequencing (NGS) of *hTERT-*repressed and segregant *hTERT-*positive 21NT-chromosome 3 hybrids may enable positional cloning of critical *hTERT* regulatory sequences.

## MATERIALS AND METHODS

### Cell culture

The mouse (A9) human chromosome 3 hybrid donor cell line A9-Hytk3 [35], carrying a selectable fusion gene marker, Hytk (Hy, bacterial hygromycin phosphotransferase; tk, herpes simplex virus thymidine kinase) tagged on the human chromosome 3 copy, was maintained in Dulbecco’s modified Eagle medium (DMEM) containing 10% foetal bovine serum (FBS; Thermo Fisher Scientific, Inc., Waltham, MA, USA) and 400 U/ml hygromycin B (Calbiochem Corp., San Diego, CA, USA). All other cell lines were grown and maintained as described previously [36]. The 21NT cell line is a primary tumour line and is considered to be an early stage in breast cancer progression [37, 38]. 21NT-exo*hTERT* (21NT cells stably overexpressing exogenous *hTERT* and referred to as PB1 from herein) were maintained in the same culture conditions as 21NT cells [23]. Our group’s previous publications [9, 19] have shown that when chromosome 3 was transferred into the cell line 21NT, down-regulation of hTERT/telomerase and senescence occurs within ∼3-4 weeks. Therefore, we have a small window in which to collect sufficient cells to perform downstream molecular analysis. To overcome this issue, we transfected the 21NT cell line with a plasmid expression vector (PCI-neo, Promega) containing the *hTERT* cDNA and picked clones (called PB1) which stably expressed exogenous *hTERT* using the CMV promoter. These PB1 cells express high levels of exogenous *hTERT* and do not go into senescence when intact whole human chromosome 3 is transferred into them [9, 19]. By expressing exogenous *hTERT* we are then able to study the repression of endogenous *hTERT* through transfection of human chromosome 3 and identify gene(s) responsible for the repression &/or de-repression of endogenous *hTERT*.

### Generation of chromosome 3 fragments and microcell-mediated monochromosome transfer (MMCT)

Microcell-mediated transfer of chromosome 3 fragments into 21NT or PB1 cells was carried out as described previously [14]. Fragmentation of chromosome 3 was achieved by exposing microcells containing monochromosome 3 to 25-50 Gy of γ-radiation prior to MMCT. Following microcell-fusion, PB1-chromosome 3 fragment hybrids were selected in medium supplemented with 400 U/ml hygromycin B. Retro-transfer of chromosome 3 fragments from PB1 hybrid clones into mouse A9 fibroblasts was conducted using the standard MMCT procedure described previously [14].

### Quantification of transcripts by quantitative real-time PCR (qRT-PCR)

Extraction of RNA from cell lines/strains was carried out using peqGOLD TriFast reagent (Peqlab; VWR International, Radnor, PA, USA) according to the manufacturer’s instructions. Removal of contaminating DNA from RNA extracts was achieved using the Deoxyribonuclease I (DNAse I), amplification grade enzyme (Invitrogen; Thermo Fisher Scientific, Inc.). Reverse transcription of 1 μg RNA into first-strand cDNA was carried out using the High Capacity cDNA Reverse Transcription kit (Applied Biosystems; Thermo Fisher Scientific, Inc.). The primer sequences and thermal cycling parameters used to quantify *hTERT, GAPDH, SETD2, BAP1, PBRM1* and *PARP-3* transcripts are listed in Table 1. qPCR was performed using iTaq™ Universal SYBR^®^ Green Supermix or Universal Probes Supermix (BioRad, Hercules, CA, USA) as described previously [36].

**Table 1:** Sequences of the synthetic oligonucleotides used as primers for qRT-PCR and thermal cycling parameters

Gene	Primer name	Sequence (5’-3’)	Primer/probe +thermal cycling parameters
***GAPDH***	GAPDH-F	GAAGGTGAAGGTCGGAGT	Primers: 0.3μM
	GAPDH R	GAAGATGGTGATGGGATTTC	Probe: 0.15μM
hTERT*	hTERT-F	GAGCTGACGTGGAAGATGAGC	Step1: 95ºC 10min
	hTERT-R	GGTGAACCTCGTAAGTTTATGCAA	Step 2: (40cycles) 95ºC 15sec, 60ºC 1min
	hTERT-Probe	6-FAM-CACGGTGATCTCTGCCTCTGCTCTCC-TAMRA	
***SETD2***	SETD2-F	ATTGAGTTTTTCTTCCTCTTGTGAGAT	
	SETD2-R	CCCAACCTAAGTTTCTGAGCTCTT	
	SETD2-Probe	6-FAM-CACATGTGGATGGCTTGCACTCATCA-MGB	
***BAP1***	BAP1-F	AGAAATACTCACCCAAGGAG	Primers: 0.45μM
	BAP1-R	TCCTTCTCTGGTCATCAATC	Step 1:
***PBRM1***	PBRM1-F	ACGGAAAATCAACATGAGTG	95ºC 30sec
	PBRM1-R	TGCCTTCATATTCTGCTTTC	Step 2: (40cycles) 95ºC 15min, 58ºC 30sec, 72ºC 15sec
***PARP3***	PARP3-F	CTGGAAAGTAAACCAAGAAGG	
	PARP3-R	TCTCTGAGGCAAAGTAGATG	

*hTERT transcripts were amplified for 50 cycles.

### Cytogenetic analysis of hybrids by fluorescence in situ hybridization (FISH)

FISH analysis was carried out as described previously [14]. A9 chromosome 3 fragment hybrids were hybridised with a total human DNA paint labelled with TexasRed, and a chromosome 3-specific green paint labelled with FITC (MetaSystems, Altlussheim, Germany).

### DNA extraction

Extraction of genomic DNA from cell lines and strains was achieved using the Wizard^®^ Genomic DNA Purification Kit (Promega, Madison, WI, USA) according to the manufacturer’s instructions.

### Sequence-tagged microsatellite mapping

PB1-chromosome 3 fragment hybrids were initially mapped as previously described [14]. Polymorphic markers spanning the whole length of human chromosome 3 were selected from the NCBI database to carry out microsatellite mapping. Due to the high level of non-informative markers in our region of interest (3p21.3-p22 and 3p12-p21.1), PB1 chromosome 3 hybrid clones were retro-transferred back into the A9 mouse background for fine-structure mapping using STS markers and standard PCR procedures.

### Gene copy number variation (CNV) analysis

CNV analysis was performed as described previously [39]. *SETD2, BAP1, PARP-3* and *PBRM1* CNV analysis was performed using pre-designed Taqman^®^ Copy Number Assay primers (Applied Biosystems; Thermo Fisher Scientific, Inc.; assay reference numbers, Hs01027663_cn, Hs02357352_cn, Hs01428519_cn and Hs06624309_cn, respectively). Assays were run in a duplex reaction with *RNAseP* Taqman^®^ Reference Assay (reference number: 4403326) to normalise target gene copy number.

### Identification of three additional candidate hTERT repressor sequences localised to human chromosome 3p21.1-3p21.3: PARP-3, BAP1 and PBRM1

Previous studies have identified three putative regions on chromosome 3p that may harbour *hTERT* repressor sequences within breast cancer cells, including 3p21.3-p22, 3p12-p21.1 and 3p21.2 [14, 19]. Therefore, a review of relevant literature was conducted in order to identify additional candidate *hTERT* repressor sequences that are (i) located within the 3p12-p22 region, (ii) have been implicated in breast cancer and (iii) have been shown to serve a functional role in the epigenetic regulation of target gene transcription through chromatin remodelling and/or have been implicated in *hTERT* transcription regulation. Three interesting candidate genes, including BRCA1 associated protein-1 (BAP1), polybromo 1 (PBRM1) and poly(ADP) ribose polymerase 3 (PARP-3) were selected for further investigation.

BAP1 has been found to interact directly with known repressors of *hTERT* transcription, including BRCA1 and E2F-1 [40–43]. Additionally, co-transfection of BAP1 and its interacting partner BRCA1, is associated with a significant reduction in the clonogenic survival of MCF7 breast cancer cells [41]. Loss of heterozygosity (LOH) of the *PBRM1* genomic locus and truncating mutations within the bromodomains (BD) of PBRM1 has been identified in breast cancer cell lines [23]. Transient overexpression of PARP-3 within the A549 lung adenocarcinoma cell line was associated with a significant reduction in telomerase activity at 48 and 96 h following transfection compared with empty vector controls [44].

### Investigating the effect of SETD2, BAP1, PARP-3 and PBRM1 overexpression on hTERT transcription within 21NT cells

The plasmid vectors pCMV6AC-*BAP1* (OriGene Technologies Inc., Rockville, MD, USA), pBABEpuro-*BAF180/PBRM1* [26], pCMVNeo-PARP-3 and associated empty vector controls were used to generate stable *BAP1, PBRM1* and *PARP-3,* 21NT transfection clones, respectively. *SETD2* and *PARP-3* cDNA was cloned into pCMVNeo vectors (OriGene Technologies, Inc.) and re-sequenced to confirm that the cDNAs were in the correct orientation. Overexpression of *SETD2, BAP1, PARP-3* and *PBRM1* within the 21NT cell line was achieved using the *Trans*IT^®^-BrCa Transfection Reagent (Mirus Bio, Madison, WI, USA) according to manufacturer’s instructions. A minimum of 5 stable 21NT-target gene and 5 stable 21NT-empty vector control clones were then isolated at random and propagated as individual cell lines. Transient overexpression of *SETD2* within 21NT cells was achieved by harvesting cells at 24 h intervals, starting from 48 h following transfection with pCMVNeo or pCMVNeo-*SETD2* plasmids. The mRNA expression levels of *SETD2, BAP1, PARP-3, PBRM1* and *hTERT* were determined as described above.

## SUPPLEMENTARY MATERIALS TABLE


