# Supplementary material for: Functional role of SETD2, BAP1, PARP-3 and PBRM1 candidate genes on the regulation of hTERT gene expression
Source: Oncotarget. 2017 Jun 27;8(37):61890–900. doi: 10.18632/oncotarget.18712 (PMC5617472; doi:10.18632/oncotarget.18712)
Supplement: Supplementary file 1 [file oncotarget-08-61890-s001.pdf]

Functional role of *SETD2, BAP1, PARP-3* and *PBRM1* candidate genes on the regulation of *hTERT* gene expression

SUPPLEMENTARY MATERIALS

Supplementary Table 1: Origin and features of each human cell line/strain utilised

Cell type	Cell line/ strain	Patient details	Cell line tissue origin	Breast cancer type	Primary tumour (PT)/ metastatic deposit (MD)	Tumorigenic (Y/N)	p53 mutation status	*HER2/ neu* positive	Molecular subtype
**Mammary epithelial cell**	**184**	F, 21 years	Breast-Reduction Mammoplasty Tissue	N/A	N/A	N/A	N/A	N/A	N/A
	**240L**	F, 19 years		N/A	N/A	N/A	N/A	N/A	N/A
	**LONZA**	F		N/A	N/A	N/A	N/A	N/A	N/A
**Breast cancer-derived**	**BT20**	C, F, 74 years	Mammary Gland	IDC	PT	Y	MUT	N	Basal A
	**BT474**	C, F, 60 years	Breast Duct	IDC	PT	Y	MUT	Y	Luminal
	**GI101**	F, 57 years	Breast Duct	IDC	PT	Y	UD	N	Basal-like
	**HCC1143**	C, F, 52 years	Breast Duct	IDC	PT	UD	MUT	N	Basal A
	**H5S78T**	C, F, 74 years	Mammary Gland	CS	PT	N	MUT	N	Basal B
	**MCF7**	C, F, 36 years	Pleural Effusion	AC	MD	N	WT	N	Luminal
	**MTSV***	-	Breast Lumen	N/A	N/A	N	N/A	N/A	N/A
	**21NT**	F, 36 years	Breast	IDC	PT	Y	MUT	Y	-
	**21MT**		Pleural Effusion	IDC	MD	Y	MUT	Y	-

The International Agency for Research on Cancer (IARC) TP53 Database (http://p53.iarc.fr/CellLines.aspx) was used to determine the p53 mutation status of breast cancer cell lines. The p53 mutation status of the H5S78T cell line was determined using the Universal Mutation Database [1]. Human epidermal growth factor receptor (HER2/neu) status and the molecular subtype of breast cancer cell lines [2, 3–4]. *SV40-immortalized breast luminal epithelial cell line. C (Caucasian), F (Female), IDC (Infiltrating/Invasive Ductal Carcinoma), CS (Carcinosarcoma), AC (Adenocarcinoma), DCIS (Ductal Carcinoma In Situ), MUT (mutation), WT (wild-type), UD (undetermined).

REFERENCES

1. Edlund K, Larsson O, Ameur A, Bunikis I, Gyllensten U, Leroy B, Sundstrom M, Micke P, Botling J, Soussi T. Data-driven unbiased curation of the TP53 tumor suppressor gene mutation database and validation by ultradeep sequencing of human tumors. Proceedings of the National Academy of Sciences of the United States of America. 2012; 109: 9551-9556.
2. Band V, Zajchowski D, Swisshelm K, Trask D, Kulesa V, Cohen C, Connolly J, Sager R. Tumor progression in four mammary epithelial cell lines derived from the same patient. Cancer Res. 1990;50:7351-7.
3. Kao J, Salari K, Bocanegra M, Choi YL, Girard L, Gandhi J, Kwei KA, Hernandez-Boussard T, Wang P, Gazdar AF, Minna JD, Pollack JR. Molecular profiling of breast cancer cell lines defines relevant tumor models and provides a resource for cancer gene discovery. PloS one. 2009; 4: e6146.
4. Hurst J, Maniar N, Tombarkiewicz J, Lucas F, Roberson C, Steplewski Z, James W, Perras J. A novel model of a metastatic human breast tumour xenograft line. British journal of cancer. 1993; 68: 274-276.
